# Interchangeability of Electrocardiography and Blood Pressure Measurement for Determining Heart Rate and Heart Rate Variability in Free-Moving Domestic Pigs in Various Behavioral Contexts

**DOI:** 10.3389/fvets.2015.00052

**Published:** 2015-11-02

**Authors:** Annika Krause, Armin Tuchscherer, Birger Puppe, Jan Langbein

**Affiliations:** ^1^Institute of Behavioural Physiology, Leibniz Institute for Farm Animal Biology (FBN), Dummerstorf, Germany; ^2^Institute of Genetics and Biometry, Leibniz Institute for Farm Animal Biology (FBN), Dummerstorf, Germany; ^3^Behavioural Sciences, Faculty of Agricultural and Environmental Sciences, University of Rostock, Rostock, Germany

**Keywords:** domestic pig, electrocardiogram, blood pressure, heart rate variability, interchangeability, Bland and Altman plot

## Abstract

This study assessed the interchangeability between heart rate (HR) and heart rate variability (HRV) measures derived from a series of interbeat intervals (IBIs) recorded via electrocardiogram (ECG) and intra-arterial blood pressure (BP) in various behavioral contexts. Five minutes of simultaneously recorded IBIs from ECG and BP signals in 11 female domestic pigs during resting, feeding, and active behavior were analyzed. Comparisons were made for measures of HR, the standard deviation of IBIs, and the root mean of the squared distances of subsequent IBIs derived from ECG and BP signals for each behavior category using statistical procedures with different explanatory power [linear regression, intraclass correlation coefficient (ICC), Bland and Altman plots, and analysis of variance (ANOVA)]. Linear regression showed a strong relationship for HR during all behaviors and for HRV during resting. Excellent ICCs [lower 95% confidence intervals (CI) >0.75] and narrow limits of agreement in all behavior categories were found for HR. ICCs for HRV reached the critical lower 95% CI value of 0.75 only during resting. Using Bland and Altman plots, HRV agreement was unacceptable for all of the behavior categories. ANOVA showed significant differences between the methods in terms of HRV. BP systematically overestimated HRV compared with ECG. Our findings reveal that HR data recorded via BP agree well those recorded using ECG independently of the activity of the subject, whereas ECG and BP cannot be used interchangeably in the context of HRV in free-moving domestic pigs.

## Introduction

Fluctuations between heartbeats in mammals are predominantly regulated by the constant interplay between the parasympathetic and sympathetic branches of the autonomic nervous system (ANS). The assessment of heart rate variability (HRV) is widely used as an indicator of autonomic function in the analysis of physiological signals in humans and animals (1–3). The mean heart rate (HR) can be interpreted as a reflection of the net effects of the interaction between both branches of the ANS. Parameters of HRV derived from cardiac interbeat interval (IBI) data provide information regarding the complex interaction between both branches (SDNN: the standard deviation of all IBIs of the data set) and regarding parasympathetic activation alone (RMSSD: the square root of the mean of the sum of the squares of differences between successive IBIs) (3).

Most research on HRV primarily occurs in humans and focuses on the relationship between autonomic functioning and diseases, such as cardiac dysfunction (4) and sudden cardiac death (5). Within the field of farm animal research, analyses of HRV have been used to investigate changes in sympathovagal balance due to pathology (6), stress (7, 8), housing and management conditions (9, 10), learning (11), pain (12, 13), and emotional states (14, 15).

Advances in telemetric technology enable the use of mobile, invasive telemetric systems that can be used to (not only) verify IBIs via the simultaneous recording of electrocardiogram (ECG) and blood pressure (BP) data in free-moving animals. This enables both the evaluation of physiological variables with a minimum of disturbance to the animal and complex experimental designs in which animals are able to range freely in groups, for example, during social interactions. In the last two decades, a number of investigations have used invasive telemetric systems in the context of pharmacological studies and cardiovascular diseases (16, 17), circadian rhythms (18), and stress (19) and were used in animals, such as rabbits (20), goats (21), monkeys (22), rats (23), dogs (24), and pigs (25). Studies using telemetry to evaluate HRV in the context of autonomic regulation usually make use of IBIs derived from ECG to calculate parameters of HRV. However, invasive telemetric technology also provides the assessment of IBIs from BP signal. One IBI is the time in milliseconds between two consecutive R-peaks in an ECG or between two consecutive systolic pressure peaks in arterial BP. As HRV may easily be biased by measurement errors in IBIs, preferably only segments of data that are free from artifacts and ectopic or anomalous beats should be included in the analysis. Additionally, it is not possible to simply omit segments of data that contain artifacts as this would interrupt the fundamental time series of the data on which the analysis is based (26, 27). Postrecording editing of the data is an indispensable procedure for the assessment of reliable data (28). As found in a recent study, the telemetric measurement of ECG in free-moving domestic pigs is likely to result in the generation of anomalous waveforms that compound the correct identification of IBIs by the software [Krause et al., under revision (29)]. Some of the errors may be explained as artifacts originating from physical activity. These movement artifacts in ECG are not present in BP signals. Therefore, BP signals are potentially useful for the collection of IBI data for further analysis of HRV, especially in experimental settings in which behaviors with an elevated level of activity, such as social interaction, are being studied. If BP signals are valid and reliable in measuring IBIs in pigs, then there are obvious potential benefits in using BP instead of ECG. To our knowledge, however, there is a lack of research on the comparability of IBI data derived from ECG and BP for measuring HR and HRV. The accuracy of BP signals in terms of HR and HRV measurement remains to be determined prior to considering any further application in the context of HRV analysis.

The aim of this study was to assess the relationship, agreement, and interchangeability between HR and HRV data derived from a time series of IBIs recorded using ECG and BP in pigs during various behavioral situations with various levels of physical activity by applying statistical methods with different explanatory powers. This would help to clarify the possibility of using data derived from BP signals in the event of data loss or a high proportion of erroneous beats in the ECG signal (or vice versa).

## Animals, Materials, and Methods

### Animals

Data from 11 female domestic pigs (*Sus scrofa*, German Landrace) from the experimental facilities for swine of the “Leibniz Institute for Farm Animal Biology in Dummerstorf” were included in the study. The pigs were housed in individual pens (2.67–3.31 m^2^) with solid and partially slatted floors and visual, olfactory, and partially tactile contact with conspecifics (snout contact) in the adjacent pen. Pigs had free access to water (nipple drinker) and were fed twice per day. The temperature was held constant at 20°C. After 1 week of acclimatization, the pigs were weighed, and their health was checked. At 11 weeks of age, the pigs underwent surgery for implantation of a telemetric device capable of recording ECG, BP, and body temperature (29). The crucial factor for the time of surgery was for the pig’s weight to exceed 30 kg, which is when the animals’ artery is sufficiently large to insert and advance the catheter inside the vessel without resistance. Prior to surgery, the pigs were handled three times daily for 1 h to socialize and habituate them to human contact and equipment monitoring.

### Instrumentation

The telemetry unit, individual receivers, data acquisition device (Power Lab), and analysis software (LabChart) were provided by Telemetry Research (Auckland, New Zealand) and ADInstruments (Oxford, UK). The implantable transmitter unit (Telemeter model TRM84PB) weighed 64 g and measured 90 mm × 45 mm × 10.5 mm. Two flexible biopotential leads (30 cm in length) and a fluid-filled catheter [1 mm OD (3Fr) Catheter, Millar Instruments, Houston, TX, USA] extended from the body of the transmitter. A temperature sensor embedded in the implant case was used to measure the animal’s body temperature. Using an individual frequency, each implanted transmitter relayed digital signals to a receiver unit attached at a 2.42-m height in front of each single housing pen. A switch at the receiver enables the transmitter to be turned on and off *in vivo* without affecting the animal. The receivers were wire connected to two digital data acquisition systems (PowerLab 16/35 and 4/35, ADInstruments) that enabled the simultaneous real-time recording of up to six pigs at a sampling rate of 2 kHz. The sampled data were stored and displayed on a PC and analyzed using LabChart Pro (Version 7.0, ADInstruments).

**Figure 1:**
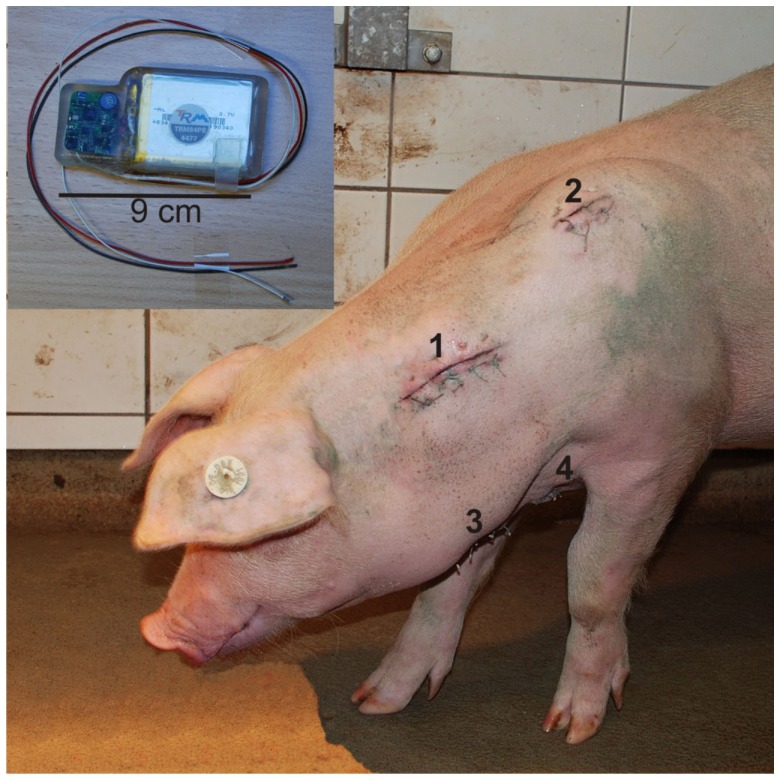
**Locations of the four components of the telemetric system exemplary for one pig 5 days after surgical procedure: positions of the transmitter body (1), positive electrode (2), catheter (3), and negative electrode (4)**. (Upper left) Transmitter body with according wires [positive electrode (black), catheter (white), and negative electrode (red)].

The pigs were fasted 12 h prior to surgery but allowed access to water. The animals were subsequently implanted with the telemetric device by the veterinarian of the institute under sterile conditions. For details regarding surgery and implantation, see Krause et al. (29). Here we give a brief summary. A subcutaneous pouch was formed at the left side of the neck for placement of the telemeter body. The pressure sensor was inserted into the left carotid artery. The negative electrode was placed lateral to the sternum, and the positive electrode was located ~2 cm lateral to the left scapula. The tips of both electrodes were enclosed by muscle tissue. Figure 1 presents the locations of the four components of the telemetric system exemplary for one pig. After completing surgery, pigs were returned to their respective home pens and allowed to recover under a heat lamp to maintain body temperature after surgery. They were observed continuously until they recovered consciousness and could stand and move safely on their own. Body temperature, ECG, and BP were monitored for 5 min every hour until the pig exhibited complete recovery from anesthesia. The pigs were administered a postsurgical medication of 50 mg/kg metamizole (Metapyrin, Serumwerk Bernburg AG, Germany) and a combination of 20 mg/kg sulfadimidine and 4 mg/kg trimethoprim (Trimethosel, Selectavet, Otto-Fischer GmbH, Weyarn-Holzolling, Germany). This regimen was continued every 24 h for 5 days postsurgery. During recovery period of 14 days, health status, behavior, impact of the transmitter and surgery on the animals, and functionality of the telemetry system were checked four times daily. Skin sutures were removed on days 10 and 11 postoperatively.

### Data Acquisition

Behavior, ECG, and BP data of 11 animals were monitored for 30 days after surgery. For the present study, data acquisition was performed on day 15 to provide sufficient time for recovery from surgery, wound healing, and examination of the compatibility of the device within the animal before data collection was started. A familiar person entered the experimental room at 8.15 a.m. and consecutively fed the animals. Both behavior and physiological responses (ECG and BP) were continuously recorded for 2 h prefeeding, during food intake and 1 h after feeding. Pig behavior was assessed using video cameras (Panasonic WV-CP500, EverFocus Endeavor SD + HD DVR) attached to each single housing pen and analyzed using the Observer XT (Version 11, Noldus, Wageningen, Netherlands). Three behavior categories, each 5 min in length, were defined: lying inactive, feeding (food intake), and active behavior (locomotion, drinking, and scratching, whereas locomotion was mostly dominated by exploration with snout contact to the ground or walls of the home pen). If one 5-min interval was continuously categorized as “resting,” “feeding,” or “active behavior,” it was chosen for further analysis or more precisely, behavioral analysis required every individual to exhibit each behavior category (resting, feeding, active) for not <5 min continuously. These different activities were chosen to assess whether agreement between the different signals was stable across a wide range of data. For each individual and behavior, one 5-min segment of IBI data from both ECG and BP signals was selected. A total of 33 5-min segments of IBI from ECG signals and 33 segments of IBI from BP signals provide the basis for all further comparisons.

Interbeat intervals derived from the ECG were calculated as the time between two consecutive R-peaks in the QRS complex, and IBIs derived from the BP signals were defined as the time between two consecutive systolic pressure peaks. QRS complexes and systolic pressure waves were automatically detected using the software. Subsequently, every 5-min segment was visually checked for correct marking. If the markings of a valid QRS complex or systolic pressure peak were missing or set falsely, they were manually adjusted using the software. If a QRS complex or systolic pressure peak was completely missing, it was corrected via interpolation calculated from the mean of three previous and three subsequent values. If more than three consecutive IBIs were missing or >5% needed interpolation, the 5-min segment was discarded from further analysis, following the recommended criteria for HRV calculations (3). HR, SDNN (an indicator of sympathetic and parasympathetic activation), and RMSSD (an indicator of parasympathetic activation) were computed by LabChart.

### Statistical Analysis

All statistical analyses were conducted utilizing SAS (Version 9.3, 2009, SAS Institute Inc., Cary, NC, USA). The normality of distribution of each parameter was assessed using a Kolmogorov–Smirnov test. Where normality assumptions were not met, data were logarithmically transformed if necessary.

A linear regression analysis was used to quantify the strength of the relationship between HR, SDNN, and RMSSD derived from the ECG and BP signals. To evaluate the agreement between the two different signals, we calculated the intraclass correlation coefficients (ICCs) and their 95% confidence intervals (CI) for each behavior category according to Shrout and Fleiss (30). ICCs >0.8 are considered to indicate good to excellent relative agreement, whereas coefficients between 0.6 and 0.8 are considered to be substantial (31, 32). Interchangeable use has been suggested to exist when the lower 95% CI value exceeds 0.75 (33). Additionally, levels of agreement between ECG and BP data were assessed using Bland and Altman plots with 95% limits of agreement (LoA) with the criteria of Altman and Bland (34). For BP being used interchangeably with ECG, 95% of the differences should fall within the LoA, and the width of LoA was also expected to be clinically acceptable. Bland and Altman plots illustrate the difference between paired observations on the *y*-axis (BP − ECG), plotted against their mean value on the *x*-axis [(BP + ECG)/2]. This provides a template with LoA and makes it possible not only to evaluate the agreement between the methods over different values of *x* but also to assess whether the range between the limits is acceptable. Furthermore, all parameters were analyzed using repeated measurements analysis of variance (ANOVA) according to the MIXED procedure included in SAS. The model included the fixed effects behavior (resting, feeding, and active), signal (ECG and BP) and behavior × signal. The analysis included the subject as a repeated factor. Least-squares means (LSM) and their standard errors (SE) were computed for each fixed effect in the models. If the fixed effect was significant, pair-wise differences of LSM were tested using the Tukey–Kramer correction. The SLICE statement of the MIXED procedure was used to conduct only specified comparisons of LSM. Effects and differences were considered significant if *P* < 0.05. Results are presented as LSM ± SE.

### Ethical Statement

All procedures involving animal handling and treatment were approved by the Committee for Animal Use and Care of the Ministry of Agriculture, Environment, and Consumer Protection of the federal state of Mecklenburg-Vorpommern, Germany (ref. no. 7221.3-1.1-037/12). After completing the entire experiment, the pigs were sedated with a combination of 2 mg/kg xylazine (Xylariem) and 20 mg/kg ketamine (Ursotamin) prior to being euthanized with an intravenous injection of 0.5 mg/kg tetracaine hydrochloride, 5 mg/kg mebezonium iodide, and 20 mg/kg embutramide (T61, Intervet International GmbH, Unterschleißheim, Germany).

## Results

The 11 pigs showed a good and uncomplicated healing process. Even after the postsurgical medication was concluded, body temperature remained constant and did not change over the 15-day observation period. Despite the stringent correction threshold of 5% and/or the requirement of missing no more than three consecutive beats, none of the 5-min segments had to be excluded from further analysis. The simultaneous measurement of BP and ECG in 11 subjects during the three different behavior categories (5 min each) provided an overall 40,124 IBIs [mean IBI count ± SD during resting = 527.5 (±69.1), during feeding = 637.2 (±48.4), and during active behavior = 659.1 (±59.3)]. In ECG, 2.5% of the IBIs needed manual correction; only 0.6% was interpolated. Regarding the BP signal, 0.3% of IBIs had to be manually corrected, and none of the beats needed interpolation.

### Linear Regression

**Figure 2:**
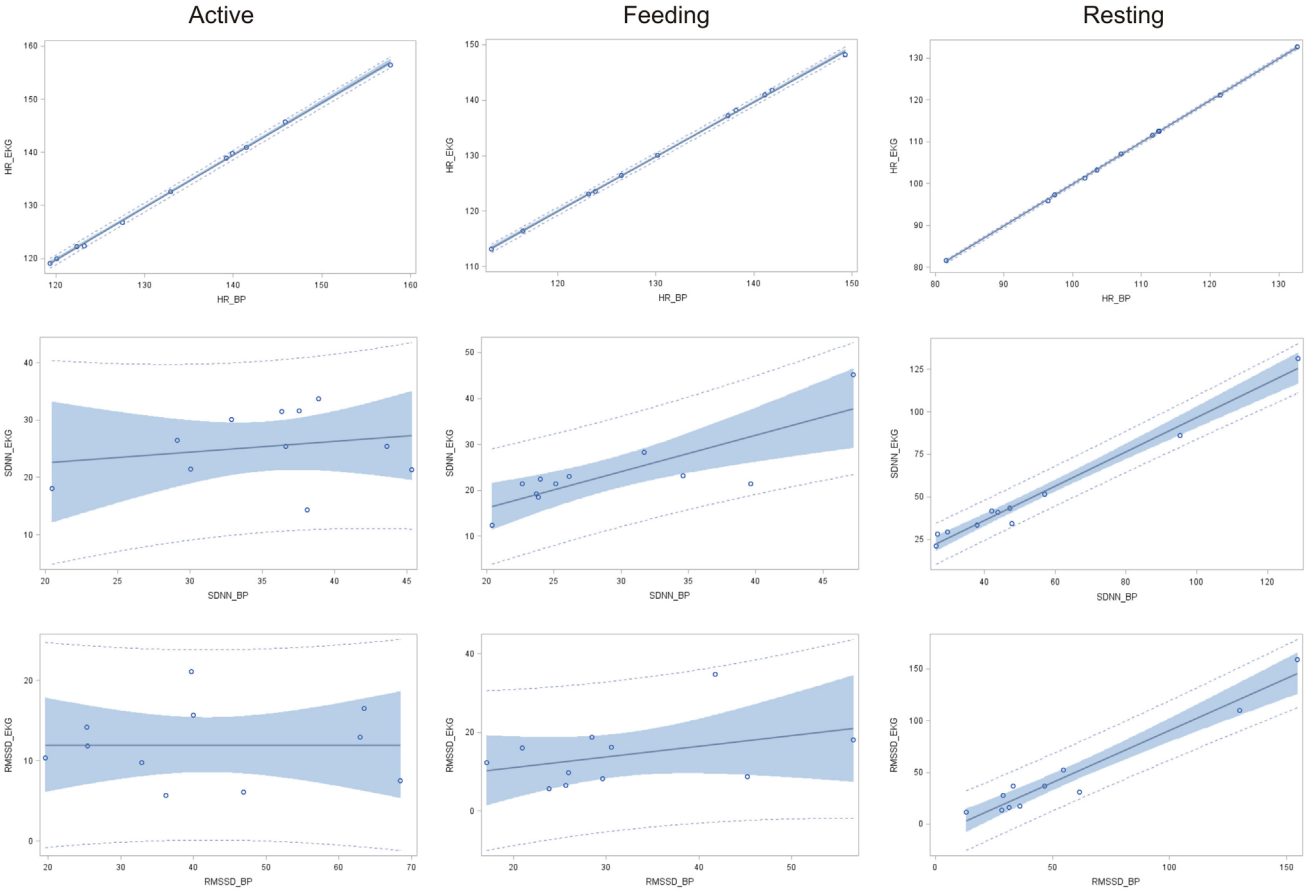
**Linear regression of HR, SDNN, and RMSSD obtained via ECG in function of those obtained using BP (*n* **=** 11) with according 95% confidence limits (gray area) and 95% prediction limits (dotted line) during active behavior, feeding, and resting**.

A significant relationship between IBIs derived from ECG and BP data was found in terms of HR for all behavior categories (resting: *r*^2^ = 0.9999, *P* < 0.001; feeding: *r*^2^ = 0.9994, *P* < 0.001; active: *r*^2^ = 0.9992, *P* < 0.001; Figure 2). The coefficient of determination for SDNN was significant only in terms of resting and feeding behavior (resting: *r*^2^ = 0.978, *P* < 0.001 and feeding: *r*^2^ = 0.656, *P* < 0.01), whereas only a weak association was found during active behavior (*r*^2^ = 0.045, *P* = 0.7). In terms of RMSSD, a strong relationship between ECG and BP data was found during resting behavior (*r*^2^ = 0.943, *P* < 0.001) but not during feeding (*r*^2^ = 0.146, *P* = 0.3) or active behavior (*r*^2^ = 0.001, *P* = 0.9).

### ICC

Intraclass correlation coefficient revealed a strong agreement between the HR data derived from ECG and BP for all behaviors (Table 1). ICC values >0.998 were obtained independent of activity level. Ninety-five percent CI for ICC varied scarcely between behavior categories, indicating that the true difference between these measurements was marginal. Notably, in terms of SDNN and RMSSD, interchangeable agreement was achieved only during resting behavior; none of the parameters reached the critical lower 95% CI value of 0.75 during feeding or active behavior.

**Table 1:** **Intraclass correlation coefficients (ICCs) of heart rate (HR), SDNN, and RMSSD derived from ECG and BP (*n* **=** 11) with 95% confidence intervals during active behavior, feeding, and resting**.

	HR	SDNN	RMSSD
Active	0.9989 (0.9976–1.0001)	0.0008^a^ (−0.5332–0.5332)	−0.0004^a^ (−0.0932–0.0932)
Feeding	0.9993 (0.9986–1.0001)	0.6027^a^ (0.2547–0.9507)	−0.0004^a^ (−0.0932–0.0932)
Resting	0.9998 (0.9997–1)	0.9821 (0.9627–1.0015)	0.9466 (0.8898–1.0034)

*^a^Denotes ICCs which do not reach a value of 0.75 at a lower 95% confidence level*.

### Bland and Altman Plots

**Figure 3:**
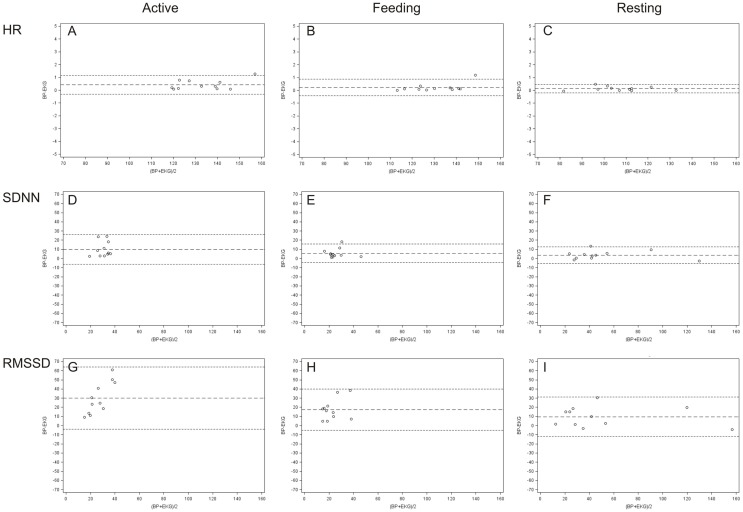
**Bland and Altman plots of differences between ECG and BP measurements in terms of HR (A–C), SDNN (D–F), and RMSSD (G–I)**. The plots illustrate, in beats per minute [HR (bpm)] and in milliseconds [SDNN and RMSSD (ms)], the differences between values derived from ECG and BP on the *y*-axis (BP–ECG) against their average values on the *x*-axis for every behavior category [active **(A,D,G)**, feeding **(B,E,H)**, and resting **(C,F,I)**].

Bland and Altman plots (35) of HR, SDNN, and RMSSD in the three behavior categories are presented in Figure 3. A narrow LoA and a small bias (mean difference) were found for the HR calculated from IBIs derived from ECG and BP with the lowest values when animals were resting. In contrast, a wider LoA and a larger bias were found for the SDNN and to an even greater extent for the RMSSD with the highest values when animals were active (Table 2). Furthermore, as can be visually observed in Figure 3, an overestimation of RMSSD and SDNN was evident when calculated from IBIs derived from BP signals.

**Table 2:** **Mean values of the differences (mean **Δ**) between HR, SDNN, and RMSSD derived from ECG and BP (BP–ECG) and the respective standard deviations (SD) of the differences, as well as 95% limits of agreement (LoA) are presented for each parameter during active behavior, feeding, and resting**.

			Active	Feeding	Resting
HR	Mean Δ		0.42	0.22	0.13
	SD		0.38	0.33	0.17
	95% LOA	Upper	1.17	0.87	0.46
		Lower	−0.32	0.25	−0.19
SDNN	Mean Δ		9.97	5.69	3.61
	SD		8.27	5.12	4.70
	95% LOA	Upper	26.18	15.73	12.82
		Lower	−6.24	−4.34	−5.61
RMSSD	Mean Δ		29.92	17.31	9.67
	SD		17.38	11.50	11.05
	95% LOA	Upper	63.99	39.85	31.33
		Lower	−4.14	−5.23	−11.98

### Analysis of Variance

**Figure 4:**
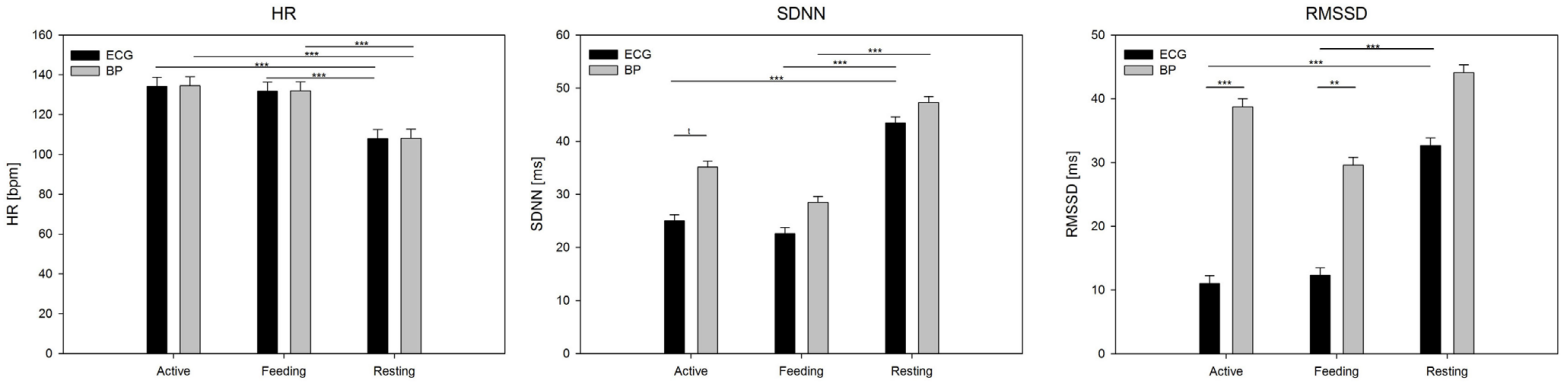
**Mean values (**±**SE) of HR, SDNN, and RMSSD derived from ECG and BP during active behavior, feeding, and resting**. Calculation was based on 5 min segments per behavior and averaged over individuals. Significance is given as follows: ***P < 0.001; **P < 0.01; *P < 0.05; and ^†^*P* < 0.1.

Analysis of variance showed a significant effect of behavior on the mean HR (*F*_2,50_ = 88.6, *P* < 0.001), but no effect of signal (*F*_1,50_ = 0.02, *P* = 0.88; Figure 4). In terms of SDNN, both signal (*F*_1,50_ = 10.7, *P* < 0.01) and behavior (*F*_2,50_ = 26.9, *P* < 0.001), but not their interaction (*P* = 0.3), had a significant impact. *Post hoc* tests revealed differences between resting and feeding behavior (*P* < 0.001) as well as between resting and active behavior (*P* < 0.001) for ECG but only between resting and feeding for the BP signal (*P* < 0.001). The most pronounced effects were found in terms of RMSSD, which was affected by signal (*F*_1,50_ = 59.8, *P* < 0.001), behavior (*F*_2,50_ = 17.1, *P* < 0.001), and their interaction (*F*_2,50_ = 7.0, *P* < 0.01). RMSSD differed significantly between the signals during feeding (*P* < 0.001) and active behavior (*P* < 0.001); however, no differences were found during resting behavior (*P* = 0.57). RMSSD derived from ECG showed significant differences between resting behavior and both feeding (*P* < 0.001) and active behavior (*P* < 0.001), whereas these effects dispersed in regards to RMSSD derived from the BP signal.

## Discussion

The current study presents the first attempt to systematically investigate the usability of intra-arterial BP for the measurement of HR and HRV in comparison to ECG in free-moving domestic pigs in order to determine whether they agree sufficiently for BP to replace ECG in experimental settings. We used various statistical procedures to assess the relationship, interchangeability, and agreement of data derived from ECG and BP signals with different explanatory power. Our findings reveal strong association and agreement between ECG and BP data in terms of HR throughout all statistical procedures with high values for coefficients in linear regression, high ICCs (>0.9) and low biases with narrow LoA in all behavior categories. Regarding HRV, linear regression at least demonstrates a connection between the signals during resting, supported by results from ICCs. An ANOVA elucidated the discrepancies between ECG and BP measurements especially in terms of RMSSD. Bland and Altman plots demonstrated differences between ECG and BP in terms of HRV; large biases and wide LoA were found independently of the activity level.

There are several theoretical advantages to the use of BP in the assessment of HR and HRV in free-moving animals. BP was found to be less susceptible to movement artifacts than ECG signals (29) whereas ECG signals are more likely to develop anomalous or ectopic beats based on the frequent biological and system constraints that may generate and perpetuate the occurrence of these artifacts in cardiac signals (26). Therefore, automatic triggering of BP (systolic pressure waves) by the software requires less manual editing of falsely triggered beats and decreases the requirement of interpolation. If BP provides results comparable to those of ECG signal in HR and HRV measures, then the goal could be to replace ECG recordings with BP recordings in the event of imprecise ECG or complete malfunction. To answer the question of whether the two methods can be used interchangeably, Lee et al. (33) proposed three criteria for agreement: first, the lower limit of the 95% CI of the ICC should be at least 0.75, second, there should be no marked systematic bias and thirdly, and there should be no statistically significant difference between mean readings obtained by the two methods. However, investigations comparing of two or more variables usually utilize linear regression analysis (35). Indeed, a strong relationship was found between ECG and BP signals across all behavioral contexts in regards to HR. The coefficient of determination is the ratio of the variation to the total variation, which indicates the percent of the data that is closest to the line of best fit. Therefore, in the case of HR, it can be deduced that the line that the points fit accounts for 99% of the variation of the points from their mean. In terms of SDNN and RMSSD, >90% of the variability was explained by the linear model only during resting behavior. Feeding and active behavior severely reduced the coefficients of determination. We can safely conclude that measurements derived from ECG and BP signals are related, at least during resting behavior. However, this does not mean that the two methods agree because *r* measures the strength of a relationship between two variables, not the agreement between them (31). A study by Lee et al. (33) advocated ICC as the optimal statistic for measuring agreement between methods. ICC reveals interchangeable use in respect to HR during all behavioral contexts. Like the regression analysis, low values were found in terms of SDNN and RMSSD for feeding and active behavior. ECG and BP may be used interchangeably for measuring HRV during resting behavior as the lower 95% CI exceeds 0.75. Bland and Altman discussed the appropriateness of ICC, in which the average correlation across all possible orderings of pairs into *x* (BP) and *y* (ECG) is a ratio of the variability between subjects to the total variability (36). The more variable the subjects are, the greater the values of ICC become. Furthermore, Müller and Büttner also illustrated the limitations in the interpretation of ICC (37). The estimates are dependent on the range of the measuring scale; the wider the range, the better the result. This may cause high ICCs during resting behavior, as the range of SDNN and RMSSD values is physiologically higher during resting than during feeding or active behavior.

Bland and Altman plots were constructed to evaluate the differences in data obtained from the same subjects using two devices (ECG and BP) that measure the same criteria (35). This alternative method meets the requirement for depending not on the range of the sample but instead considering the differences between the measurements for each subject. The mean difference (bias) and the standard deviation of the differences enable the calculation of the size of the difference that is likely to arise between the two methods. Regarding HR, we found a mean difference of 0.26 bpm, and 95% of the differences lay between −0.37 and 0.89 bpm (LoA). Thus, it is unlikely (a probability of <0.05) that measurements using the two methods would differ by >1.26 bpm. The two methods could be used interchangeably if differences in measurements of this order did not matter. How far apart measurements can be without inducing difficulties is a question of judgment (34). If this is not sufficient to cause problems in interpretation, the old method can be replaced by the new or both can be used interchangeably. Regarding HR, differences between the measurements in all behavior categories were found to be acceptable, and the largest absolute bias of 0.42 bpm was found during active behavior. This value may be negligible for non-medical purposes in future research. In contrast, SDNN and RMSSD showed the greatest biases during active behavior, with correspondingly wide LoA. These values were not considered appropriate. Biases of 3.61 ms (SDNN) and 9.67 ms (RMSSD) during resting behavior were also not considered reasonable because these values may lead to the misinterpretation of HRV measurements in the context of autonomic control and balance. Noticeably, Bland and Altman plots illustrated a systematic overestimation of HRV parameters by BP signals throughout all behavior categories with the most pronounced effect on RMSSD. This overestimation was underpinned by the ANOVA results, which showed that the calculated HRV parameters from the two different signals consequently led to differing results within the various behavior categories. SDNN, which reflects long-term fluctuations in IBIs, was found to differ between active and resting as well as between feeding and resting, if the data were derived from ECG. Regarding SDNN derived from BP, only differences between feeding and resting were found. This effect becomes more apparent in RMSSD, which reflects short-term fluctuations in IBIs. The effect of behavior was completely absent from the BP signals, as RMSSD was not found to differ between active, feeding, or resting, whereas differences between these behaviors were found in RMSSD derived from the ECG signals.

Summarizing the results of the different statistical procedures, we conclude that ECG and BP may be used interchangeably to calculate HR; however, this is not the case for HRV measurements. This effect may be caused by the morphology of the BP signal. ECG provides very sharp and thus clearly recognizable peaks (R-waves in QRS complex), which facilitates exact peak definition. In contrast, the BP signal yields systolic pressure waves, which are substantially flatter and platykurtic compared with ECG waves. This may provide a further scope of triggering and may result in greater variation of the distances between successive IBIs. In the case of HR, this may not have consequences, as HR is measured using the mean number of beats per minute and, thus, the variance of single values is leveled. In contrast, in the context of HRV measurements, this inaccuracy in triggering leads to greater values throughout the measurement. This effect is more pronounced in RMSSD, because this short-term fluctuation is calculated from beat-to-beat variability, whereas SDNN reflects the deviation of all registered IBI within the 5-min segment. As a consequence, SDNN is less altered by this effect than RMSSD. Furthermore, a generally lower HRV is apparent during feeding and active behavior. Therefore, aberrations of trigger points in the BP signal may be more weighted in this case due to the smaller range in which variability is measured.

Overall, BP and BP variability are indispensable tools for the investigation of sympathetic activation which is not possible by measuring HRV alone. If ECG was found to be replaceable by BP in terms of HRV measurement, BP alone could be used as source for the investigation of autonomic regulation. This beneficial value could have found application in future research using non-invasive BP methods (e.g., ear worn sensors or tail cuffs). However, this study shows that ECG might be replaceable by BP recordings in terms of HR and potentially for HRV during resting conditions, but BP could not reliably be used in place of ECG in terms of HRV during feeding or active behavior in domestic pigs.

## Conclusion

Considering the high values for regression and ICC as well as narrow LoA and the lack of relevant bias in the Bland and Altman plots, our results suggest, on the one hand, very good to excellent agreement between the ECG and BP recordings in terms of HR. Both signals can be considered interchangeable across all behavior categories. On the other hand, in the context of HRV, a lack of relationship and agreement between both signals during feeding and active behavior was demonstrated using all statistical approaches. High values for regression and ICC were found only during resting. This supported interchangeable use at least during resting behavior. However, large biases with accordingly wide LoA in terms of SDNN and RMSSD during resting were considered unacceptable for our purposes. We conclude that HR values derived from BP agreed well with those derived from ECG independently of the activity of the subject. Additionally, ECG and BP should not be used interchangeably in the context of HRV in free-moving domestic pigs. These findings that HRV may not readily be determinable by using BP signal contribute to the validity and application of HRV measurement in future studies, such as in the field of Animal Welfare Research.

## Conflict of Interest Statement

None of the authors in this paper have a financial or personal relationship with other people or organizations that could inappropriately influence or bias the content of the paper.

## References

[1] Malik M Camm AJ Heart Rate Variability. New York, NY: Futura Publishing Company Inc (1995).

[2] Task Force of the European Society of Cardiology; North American Society of Pacing and Electrophysiology. Heart rate variability: standards of measurement, physiological interpretation, and clinical use. Circulation (1996) 93:1043–65. 10.1161/01.CIR.93.5.1043 8598068

[3] Von Borell E Langbein J Després G Hansen S Leterrier C Marchant-Forde JN Heart rate variability as a measure of autonomic regulation of cardiac activity for assessing stress and welfare in farm animals – a review. Physiol Behav (2007) 92:293–316. 10.1016/j.physbeh.2007.01.007 17320122

[4] Flynn AC Jelinek HF Smith M. Heart rate variability analysis: a useful assessment tool for diabetes associated cardiac dysfunction in rural and remote areas. Aust J Rural Health (2005) 13:77–82. 10.1111/j.1440-1854.2005.00658.x 15804330

[5] Lammers A Kämmerer H Hollweck R Schneider R Barthel P Braun S Impaired cardiac autonomic nervous activity predicts sudden cardiac death in patients with operated and unoperated congenital cardiac disease. J Thorac Cardiovasc Surg (2006) 132:647–65. 10.1016/j.jtcvs.2006.03.057 16935122

[6] Nolan J Batin PD Andrews R Lindsay SJ Brooksby P Mullen H Prospective study of heart rate variability and mortality in chronic heart failure – results of the United Kingdom heart failure evaluation and assessment of risk trial (UK-heart). Circulation (1998) 98:1510–6. 10.1161/01.CIR.98.15.1510 9769304

[7] Mohr E Langbein J Nürnberg G Heart rate variability – a noninvasive approach to measure stress in calves and cows. Physiol Behav (2002) 75:251–9. 10.1016/S0031-9384(01)00651-5 11890975

[8] de Jong IC Sgoifo A Lambooij E Korte SM Blokhuis HJ Koolhaas JM Effects of social stress on heart rate and heart rate variability in growing pigs. Can J Anim Sci (2000) 80:273–80. 10.4141/A99-085

[9] Baymann U Langbein J Siebert K Nürnberg G Manteuffel G Mohr E Cognitive enrichment in farm animals – the impact of social rank and social environment on learning behaviour of dwarf goats. Berl Munch Tierarztl (2007) 120:89–97. 10.2376/0005-9366-120-89 17416130

[10] Hagen K Langbein J Schmied C Lexer D Waiblinger S. Heart rate variability in dairy cows – influences of breed and milking system. Physiol Behav (2005) 85:195–204. 10.1016/j.physbeh.2005.03.019 15894344

[11] Langbein J Nürnberg G Manteuffel G. Visual discrimination learning in dwarf goats and associated changes in heart rate and heart rate variability. Physiol Behav (2004) 82:601–9. 10.1016/j.physbeh.2004.05.007 15327907

[12] Rietmann TR Stauffacher M Bernasconi P Auer JA Weishaupt MA. The association between heart rate, heart rate variability, endocrine and behavioural pain measures in horses suffering from laminitis. J Vet Med A Physiol Pathol Clin Med (2004) 51:218–25. 10.1111/j.1439-0442.2004.00627.x 15315700

[13] Halmer C Franken K Licka T Correlation of electrocardiographic parameters (heart rate variability and heart rate) with Obel grading of pain of horses with laminitis. Pferdeheilkunde (2014) 30:140–7.

[14] Désiré L Veissier I Desprès G Boissy A. On the way to assess emotions in animals: do lambs (Ovis aries) evaluate an event through its suddenness, novelty, or unpredictability? J Comp Psychol (2004) 118:363–74. 10.1037/0735-7036.118.4.363 15584773

[15] Zebunke M Puppe B Langbein J. Effects of cognitive enrichment on behavioural and physiological reactions of pigs. Physiol Behav (2013) 118:70–9. 10.1016/j.physbeh.2013.05.005 23680428

[16] Gelzer ARM Ball HA. Validation of a telemetry system for measurement of blood pressure, electrocardiogram and locomotor activity in beagle dogs. Clin Exp Hypertens (1997) 19:1135–60. 10.3109/10641969709083209 9310208

[17] Gross V Plehm R Tank J Jordan J Diedrich A Obst M Heart rate variability and baroreflex function in AT(2) receptor-disrupted mice. Hypertension (2002) 40:207–13. 10.1161/01.HYP.0000027279.69240.75 12154115

[18] Janssen BJA Leenders PJA Smits JFM. Short-term and long-term blood pressure and heart rate variability in the mouse. Am J Physiol Regul Integr Comp Physiol (2000) 278:R215–25. 10644642 10.1152/ajpregu.2000.278.1.R215

[19] Farah VMA Joaquim LF Bernatova I Morris M. Acute and chronic stress influence blood pressure variability in mice. Physiol Behav (2004) 83:135–42. 10.1016/j.physbeh.2004.08.004 15501500

[20] Van den Buuse M Malpas SC. 24-hour recordings of blood pressure, heart rate and behavioural activity in rabbits by radio-telemetry: effects of feeding and hypertension. Physiol Behav (1997) 62:83–9. 10.1016/S0031-9384(97)00145-5 9226346

[21] Hydbring E Cvek K Olsson K Telemetric registration of heart rate and blood pressure in the same unrestrained goats during pregnancy, lactation and the nonpregnant, nonlactating period. Acta Physiol Scand (1999) 165:135–41. 10.1046/j.1365-201x.1999.00498.x 10090324

[22] Chaves AA Keller WJ O’Sullivan S Williams MA Fitzgerald LE McPherson HE Cardiovascular monkey telemetry: sensitivity to detect QT interval prolongation. J Pharmacol Toxicol Methods (2006) 54:150–8. 10.1016/j.vascn.2006.03.004 16679034

[23] Beig MI Bhagat N Talwar A Chandra R Fahim M Katyal A. Simultaneous recording of electroencephalogram and blood pressure in conscious telemetered rats during ictal state. J Pharmacol Toxicol Methods (2007) 56:51–7. 10.1016/j.vascn.2006.12.006 17336099

[24] Ollerstam A Visser SAG Duker G Forsberg T Persson AH Nilsson LB Comparison of the QT interval response during sinus and paced rhythm in conscious and anesthetized beagle dogs. J Pharmacol Toxicol Methods (2007) 56:131–44. 10.1016/j.vascn.2007.05.002 17689270

[25] Poletto R Janczak AM Marchant-Forde RM Marchant-Forde JN Matthews DL Dowell CA Identification of low and high frequency ranges for heart rate variability and blood pressure variability analyses using pharmacological autonomic blockade with atropine and propranolol in swine. Physiol Behav (2011) 103:188–96. 10.1016/j.physbeh.2011.01.019 21281655

[26] Kamath MV Fallen EL Correction of the heart rate variability signal for ectopics and missing beats. In: Malik M Camm AJ, editors. Heart Rate Variability. Armonk, NY: Futura Publishing Company (1995). p. 75–85.

[27] Berntson GG Bigger T Jr Eckberg DL Grossman P Kaufmann PG Malik M Heart rate variability: origins methods, and interpretive caveats. Psychophysiology (1997) 34:623–48. 10.1111/j.1469-8986.1997.tb02140.x 9401419

[28] Marchant-Forde RM Marchant-Forde JN. Pregnancy-related changes in behavior and cardiac activity in primiparous pigs. Physiol Behav (2004) 82:815–25. 10.1016/j.physbeh.2004.06.021 15451645

[29] Krause A Zebunke M Bellmann O Mohr E Langbein J Puppe B Surgical implantation and functional assessment of an invasive telemetric system to measure autonomic responses in domestic pigs. Vet J (in press). 10.1016/j.tvjl.2015.10.050 26626089

[30] Shrout PE Fleiss JL. Intraclass correlations: uses in assessing rater reliability. Psychol Bull (1979) 86:420–8. 10.1037/0033-2909.86.2.420 18839484

[31] Nunan D Jakovljevic DG Donovan G Hodges LD Sandercock GRH Brodie DA. Levels of agreement for RR intervals and short-term heart rate variability obtained from the polar S810 and an alternative system. Eur J Appl Physiol (2008) 103:529–37. 10.1007/s00421-008-0742-6 18427831

[32] Pinna GD Maestri R Torunski A Danilowicz-Szymanowicz L Szwoch M LaRovere MT Heart rate variability measures: a fresh look at reliability. Clin Sci (2007) 113:131–40. 10.1042/CS20070055 17381425

[33] Lee J Koh D Ong CN. Statistical evaluation of agreement between two methods for measuring a quantitative variable. Comput Biol Med (1989) 19:61–70. 10.1016/0010-4825(89)90036-X 2917462

[34] Altman DG Bland JM Measurement in medicine: the analysis of method comparison studies. Statistician (1983) 32:307–17. 10.2307/2987937

[35] Bland JM Altman DG. Statistical methods for assessing agreement between two methods of clinical measurement. Lancet (1986) 1:307–10. 10.1016/S0140-6736(86)90837-8 2868172

[36] Bland JM Altman DG. A note on the use of the intraclass correlation coefficient in the evaluation of agreement between two methods of measurement. Comput Biol Med (1990) 20:337–40. 10.1016/0010-4825(90)90013-F 2257734

[37] Müller R Büttner P A critical discussion of intraclass correlation coefficients. Stat Med (1997) 16:821–2. 7701147 10.1002/sim.4780132310

